# Determinants of mastitis in women in the CASTLE study: a cohort study

**DOI:** 10.1186/s12875-015-0396-5

**Published:** 2015-12-16

**Authors:** Meabh Cullinane, Lisa H. Amir, Susan M. Donath, Suzanne M. Garland, Sepehr N. Tabrizi, Matthew S. Payne, Catherine M. Bennett

**Affiliations:** Judith Lumley Centre (formerly Mother & Child Health Research), La Trobe University, Melbourne, VIC 3000 Australia; Murdoch Childrens Research Institute, The Royal Children’s Hospital, Parkville, VIC 3052 Australia; University of Melbourne Department of Paediatrics, The Royal Children’s Hospital, Parkville, VIC 3052 Australia; Women’s Centre for Infectious Diseases, Royal Women’s Hospital, Parkville, VIC 3052 Australia; University of Melbourne Department of Obstetrics and Gynaecology, The Royal Women’s Hospital, Parkville, VIC 3052 Australia; School of Women’s and Infants’ Health, University of Western Australia, Crawley, WA Australia; Centre for Population Health Research, Deakin University, Burwood, VIC 3125 Australia

**Keywords:** Breastfeeding, Mastitis, Breast infection, *Staphylococcus aureus*

## Abstract

**Background:**

Mastitis is an acute, debilitating condition that occurs in approximately 20 % of breastfeeding women who experience a red, painful breast with fever. This paper describes the factors correlated with mastitis and investigates the presence of *Staphylococcus aureus* in women who participated in the CASTLE (Candida and Staphylococcus Transmission: Longitudinal Evaluation) study. The CASTLE study was a prospective cohort study which recruited nulliparous women in late pregnancy in two maternity hospitals in Melbourne, Australia in 2009–2011.

**Methods:**

Women completed questionnaires at recruitment and six time-points in the first eight weeks postpartum. Postpartum questionnaires asked about incidences of mastitis, nipple damage, milk supply, expressing practices and breastfeeding problems. Nasal and nipple swabs were collected from mothers and babies, as well as breast milk samples. All samples were cultured for *S. aureus*. “Time at risk” of mastitis was defined as days between birth and first occurrence of mastitis (for women who developed mastitis) and days between birth and the last study time-point (for women who did not develop mastitis). Risk factors for incidence of mastitis occurring during the time at risk (Incident Rate Ratios [IRR]) were investigated using a discrete version of the multivariable proportional hazards regression model.

**Results:**

Twenty percent (70/346) of participants developed mastitis. Women had an increased risk of developing mastitis if they reported nipple damage (IRR 2.17, 95 % CI 1.21, 3.91), over-supply of breast milk (IRR 2.60, 95 % CI 1.58, 4.29), nipple shield use (IRR 2.93, 95 % CI 1.72, 5.01) or expressing several times a day (IRR 1.64, 95 % CI 1.01, 2.68). The presence of *S. aureus* on the nipple (IRR 1.72, 95 % CI 1.04, 2.85) or in milk (IRR 1.78, 95 % CI 1.08, 2.92) also increased the risk of developing mastitis.

**Conclusions:**

Nipple damage, over-supply of breast milk, use of nipple shields and the presence of *S. aureus* on the nipple or in breast milk increased the mastitis risk in our prospective cohort study sample. Reducing nipple damage may help reduce maternal breast infections.

## Background

Mastitis is an acute, debilitating condition that occurs in approximately 20 % of breastfeeding women who experience a red, painful breast with fever [1–3]. It is a distressing condition which negatively impacts the daily activities of sufferers [4, 5]. Most episodes of mastitis occur in the first two months postpartum [1–3] and recurrence rates of up to about 10 % have been reported [2, 3, 6]. Early management of mastitis involves general measures to improve drainage of the breast and reduce inflammation [7]. Medical management by general practitioners in the community usually involves oral antibiotics [8], with minimal interference to breastfeeding, however some women require hospitalisation or develop a breast abscess [9]. An earlier Melbourne-based study found approximately 3 % of mastitis sufferers developed a breast abscess subsequent to a mastitis episode [10].

Nipple pain and damage, maternal stress and fatigue, attachment difficulties, milk stasis and restrictions from a tight fitting bra have been implicated as contributing to mastitis [1, 3, 11–14]. Whereas some studies have found that women with mastitis are not more or less likely to continue breastfeeding than other women [1], others have found that mastitis sufferers are more likely to breastfeed for longer than women without mastitis [15, 16]. There have been reports that women receiving private or birth centre care are also more likely to develop mastitis than other women [1, 17, 18]. Anecdotally breast milk expression has been linked to mastitis, and expressing breast milk has become more prevalent, even for healthy term infants [19, 20], however this has not been fully investigated. A recent study using a case-control design found an association between using breast pumps and mastitis, but questionnaire data were collected retrospectively, not at the time of mastitis, so it is unclear if the expressing was a risk for or a consequence of mastitis [21].

Traditionally, *Staphylococcus aureus* has been considered the most common aetiological agent of mastitis and is frequently isolated in cases of infective mastitis and breast abscesses [22, 23]. In a case-control study, Amir *et al.* found that women with mastitis were more likely to have *S. aureus* present in breast milk than women in the control group [24]. At present, the role of other organisms such as coagulase-negative *S. aureus* is unclear [25, 26].

The aims of this prospective study are to describe the incidence and correlates of mastitis in breastfeeding women within the first two months after birth. We also investigated the presence of *S. aureus* in milk during mastitis episodes compared to milk samples collected from healthy women during the study.

## Methods

### Sample

The CASTLE (Candida and Staphylococcus Transmission: Longitudinal Evaluation) study investigated the microorganisms involved in the development of mastitis and “breast thrush” among breastfeeding women [27], and confirmed the role of *Candida* species in the symptoms of “breast thrush” [28]. Three hundred and sixty nulliparous women were recruited from two hospitals in Melbourne, Australia: the Royal Women’s Hospital, (RWH) a public tertiary referral centre, and Frances Perry House (FPH), a private co-located hospital. At recruitment participants completed a questionnaire asking about maternal age, gestational age, intended length of breastfeeding duration, highest level of educational attainment, marital status and previous staphylococcal infections. Following birth, participants were followed-up six times: in hospital and then at home weekly until four weeks postpartum and at eight weeks by telephone.

Eligibility criteria for the study were: between 18–50 years of age; nulliparity; ≥ 36 weeks pregnant at recruitment; singleton pregnancy; breastfeeding intention for at least eight weeks postpartum; sufficient proficiency in English to complete written questionnaires and a telephone interview; residing ≤ 16 km from Melbourne Central Business District (CBD). Criteria for exclusion were: medical conditions which do not allow breastfeeding; breast reduction surgery; dermatitis on nipple during pregnancy; under care of the Women’s Alcohol and Drug Service (WADS); under care of Mental Health Service or social worker.

### Data measures

#### Questionnaires

Self-administered questionnaires completed in hospital after birth and at weeks 1, 2, 3, 4 and 8 postpartum collected information about milk supply, nipple damage and mastitis. At weeks 1, 2, 3, 4 and 8 postpartum women were asked to rate their milk supply since their last interview. They could state that they were “producing enough milk for their baby”, “not producing enough milk”, “over-producing breast milk” or that their “milk supply varied”. They were also asked: “In the last week, have you been expressing breast milk?” The women who had expressed in the last week were asked “on average, how often do you express?” and could select one of the following options: “once this week”, “several times this week”, “once a day”; “several times a day” or “it varies” [29].

At all time-points postpartum, participants were asked whether they had any nipple damage. If they had, they could define this damage as a small graze/crack (<2 mm in length), a moderate graze/crack (2 to 9 mm in length) or a severe graze/crack (≥10 mm &/or yellow colour present).

Participants were given the opportunity to report any problems they had with breastfeeding. At weeks 1, 2, 3, 4 and 8 postpartum women were asked whether they had any problems with breastfeeding since their last interview. They were supplied with a list of problems, including attachment issues, over or under-supply of breast milk, use of nipple shield to feed, having an unsettled infant, or an infant not interested in feeding. Women were asked about nipple pain and whether they had used any creams or ointments on their nipples, including use of hydrated polymer (hydrogel) dressings (results have been published separately) [30, 31]. They could also state a breastfeeding problem which was not listed.

There is no standard way of diagnosing mastitis. Therefore, we asked about a range of breast symptoms and associated fever or flu-like symptoms, as has been used previously [1, 32]. For this analysis the definition of mastitis was the development of at least two breast symptoms (pain, redness, lump) and at least one systemic symptom (fever or flu-like symptoms) [1, 32] or treatment of mastitis with antibiotics. Women were asked to rate how mastitis had interfered with breastfeeding and with activities of daily living on a scale of 0 to 4 where 0 was “no interference” and 4 was “quite a bit of interference” [5].

If a participant ceased breastfeeding during the first four weeks postpartum, the subsequent home visits were not conducted but the participant was contacted at eight weeks to complete the final questionnaire by telephone.

#### Microbiology

Women provided nasal and nipple (both nipples) swabs and breast milk samples (both breasts). Nasal and oral swabs were obtained from infants. In addition, all women were given a 70 ml sterile container (Sarstedt Australia Pty. Ltd.) and asked to collect a sample of milk from the affected breast should they develop mastitis during the first eight weeks postpartum between their scheduled weekly visits. Samples were collected and cultivated for *S. aureus* as described in the study protocol [27].

### Data analysis

Descriptive analysis included maternal demographic characteristics and details about the birth, breastfeeding duration, breastfeeding difficulties, and episodes of mastitis. Using information from the successive postpartum time-points, we investigated possible predictors of (or risk factors for) mastitis incidence. We defined “time at risk” of mastitis as days between birth and first occurrence of mastitis (for women who developed mastitis) and days between birth and the last study time-point (for women who did not develop mastitis). We used a discrete version of the multivariate proportional hazards regression model [33] to investigate whether mothers’ reported nipple damage, “over-production of milk”, breastfeeding attachment problems, use of nipple shield to feed, milk expressing, use of hydrogel dressings, and presence of *S. aureus* in samples collected from swabs and breast milk samples were risk factors for mastitis incidence if they occurred during the time at risk. For each of the risk factors investigated (mothers’ reported nipple damage, “over-production of milk”, etc), we estimated an incidence rate ratio: the incidence rate of mastitis in those WITH the risk factor during the “time at risk” divided by the incidence rate of mastitis in those WITHOUT the risk factor during the “time at risk”. We used the glm command in Stata with link function = cloglog, family = binomial to estimate the rate ratios. This method uses time at risk and time-varying risk factors. Comparisons are presented using incident rate ratios, 95 % confidence intervals (CIs), and *p*-values. All analyses were carried out using Stata 13 software [34].

### Ethics approval

The study received approval from La Trobe University Human Ethics Committee (LTU UHEC No. 06-078), the RWH Human Research Ethics Committee (RWH HREC No. 06-41) and the Medical Advisory Committee at FPH. All participants provided written informed consent.

## Results

### Sample

Three hundred and sixty women were recruited to the CASTLE study between October 2009 and May 2011. During the study, 14 women withdrew after giving birth, leaving 346 women available for data collection (survey results and microbiological data) at defined time-points postpartum.

Information about the main socio-demographic characteristics of the sample is shown in Table 1. Overall, the women’s mean age was 32.7, with a range of between 19 and 44 years. Seventy seven percent of participants (*n* = 267) held a University degree or higher, indicating that this cohort represents a highly educated sample of the general population. Breastfeeding intention ranged from one month to 24 months, with a mean of ten months. The method of birth was Caesarean section for 45 % of study participants. Ten percent of participants (*n* = 36) reported suffering from previous staphylococcal infections, such as boils, abscesses or sores inside the nose.

**Table 1:** Characteristics of CASTLE study participants

Maternal characteristics (*n* = 346)	*n*	Percent
Hospital		
Royal Women’s Hospital (public)	154	44.5
Frances Perry House (private)	192	55.5
Age (years - mean, SD, range)	32.7 (4.1, 19 - 44)	
Education level		
Tertiary degree or higher	267	77
Other	79	23
Breastfeeding intention (months - mean, range)	9.7 (1 - 24)	
Previous staphylococcal infections (e.g. boils, abscesses, sores inside the nose)		
Yes	284	82.1
No	36	10.4
Unsure	26	7.5
Caesarean birth	156	45

### Incidence of mastitis

**Fig. 1:**
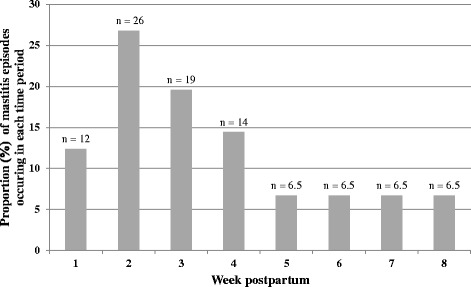
Proportion (%) of mastitis episodes per week in women in the CASTLE study (*n* = 97 mastitis episodes in 70 women). Mastitis episodes in weeks 5 to 8 were averaged; 24 women experienced mastitis in this time period

Seventy women experienced mastitis, representing 20 % of participants (70/346). There was no difference in the prevalence of mastitis among public and private patients; (40/192 private patients, 30/124 public patients experienced mastitis). There were 97 episodes of mastitis in total. Figure 1 shows the proportion (%) of mastitis episodes per week. Because women were contacted at eight weeks postpartum and asked about mastitis episodes they experienced since their last home visit at week 4, mastitis episodes experienced by women in weeks 5 to 8 were averaged. Twenty four women experienced mastitis in this time period. The majority of mastitis episodes (71 episodes, 73 % of the total) occurred in the first four weeks postpartum. Forty eight women experienced mastitis once, while 17 and 5 women experienced this condition two and three times respectively.

Mastitis sufferers were asked to rate how mastitis had interfered with both breastfeeding and with activities of daily living on a scale of 0 (no interference) to 4 (quite a bit of interference). Fifty percent (35/70) of women with mastitis reported that it interfered with breastfeeding (scored 3 or 4 on a 4-point scale) and 60 % (42/70) of women with mastitis reported that it interfered with activities of daily living (scale as above).

### Correlates of mastitis

The total “time at risk” (i.e. from birth to first mastitis or last interview postpartum if no mastitis) was fifty person-years. Potential correlates of mastitis incidence were investigated using information obtained from women at each time-point on (i) their expressing habits, (ii) self-reported breastfeeding problems and (iii) whether *S. aureus* was isolated at each time-point in nipple and milk samples from women or oral and nasal samples from infants (Table 2). Specific potential determinants of mastitis investigated were: nipple damage; expressing several times per day; attachment problems; over-supply of breast milk; use of nipple shields; use of hydrogel dressings; *S.aureus* cultured from nipple swabs collected from hospital onwards; *S. aureus* cultured from milk samples from hospital onwards and *S. aureus* cultured from the infant (either from oral or nasal swabs) from hospital onwards.

**Table 2:** Correlates of mastitis incidence in women in the CASTLE study (n =346; 70 women with mastitis)

Correlate (occurring from hospital onwards)		Number of women with at least one mastitis event	Person-years at risk of first mastitis event	Estimated Incidence Rate Ratio (IRR)	95 % CI for IRR	*p value*
Any nipple damage	yes	55	31.8	2.17	1.21, 3.91	0.01
	no	15	18.3			
“Over-producing milk”	yes	26	9.7	2.60	1.58, 4.29	<0.005
	no	44	40.4			
Attachment problem	yes	45	24.3	1.96	1.18, 3.24	0.009
	no	25	25.8			
Use of nipple shield	yes	23	7.4	2.93	1.72, 5.01	<0.005
	no	47	42.7			
Expressing several times a day	yes	33	17.7	1.64	1.01, 2.68	0.047
	no	37	32.4			
Use of hydrogel dressings	yes	15	7.0	1.72	0.96, 3.09	0.071
	no	55	43.0			
*S. aureus* isolated from nipple	yes	46	26.5	1.72	1.04, 2.85	0.035
	no	24	23.5			
*S. aureus* isolated from milk	yes	44	24.6	1.78	1.08, 2.92	0.023
	no	26	25.4			
*S. aureus* isolated from infants	yes	55	34.1	1.74	0.97, 3.11	0.062
	no	15	15.9			

**Fig. 2:**
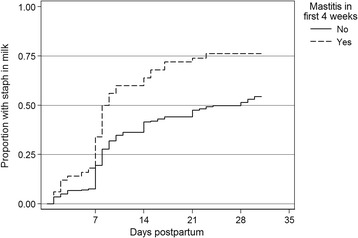
Time to first isolation of *Staphylococcus aureus* in milk (following hospital)

**Fig. 3:**
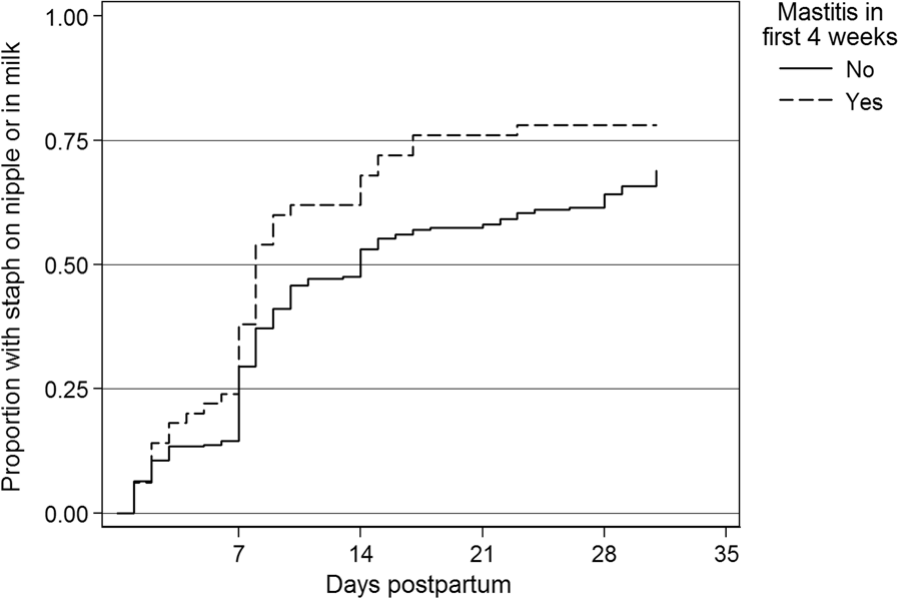
Time to first isolation of *Staphylococcus aureus* on nipple or in milk (following hospital)

At each time-point in the first 4 weeks postpartum, women who had already reported any nipple damage had a more than two-fold increased risk of developing mastitis compared to women without nipple damage (Incident Rate Ratio (IRR) 2.17, 95 % CI 1.21, 3.91). Similarly, women reporting an over-supply of breast milk, attachment problems, nipple shield use or who expressed several times a day had a significantly increased risk of developing mastitis compared to women who did not report these (Table 2). However, women who reported any expressing in the week(s) before questionnaire administration did not have an increased risk of developing mastitis (IRR 1.35, 95 % CI 0.70, 2.57). Mothers with *S. aureus* isolated from their nipple and/or breast milk had an increased risk of subsequently developing mastitis (nipple IRR 1.72, 95 % CI 1.04, 2.85, milk IRR 1.78, 95 % CI 1.08, 2.92). The proportion of women who had *S. aureus* isolated in their milk (Fig. 2) or nipple or milk (Fig. 3), are shown at weeks 1, 2, 3 and 4. Women who developed mastitis in the first 4 weeks were more likely to have *S. aureus* isolated at each visit after week 1. There was some evidence to suggest that use of hydrogel dressings or *S. aureus* isolation from either infant nasal or oral swabs led to an increased risk of developing mastitis (hydrogel dressing use: IRR = 1.7; 95 % CI 0.96, 3.09; *S. aureus* isolation from either infant nasal or oral swabs IRR 1.74, 95 % CI 0.97, 3.11). However, maternal nasal carriage of *S. aureus* did not increase the risk of risk of developing mastitis (IRR 1.11, 95 % CI 0.68, 1.81).

### *S. aureus* in breast milk samples

Breast milk samples were collected from 20 women who reported mastitis at the time of collection or the day before milk collection. Because mastitis could be present in one breast or both breasts, in total 27 mastitis milk samples were collected from these 20 women (Table 3). Fifty nine percent (16/27) of these milk samples were culture-positive for *S. aureus* (95 % CI 39 %, 78 %).

**Table 3:** Milk samples collected in women with and without symptoms of mastitis in which *S. aureus* was isolated (total number of women = 346)

*S. aureus*-positive milk samples^a^	*n*	% (95 % CI)
Women with mastitis (*n* = 27)^b^	16	59.3 (39, 78)
Women without mastitis:		
Week 1 (*n* = 657)	207	31.5 (28, 35)
Week 2 (*n* = 660)	172	26.1 (23, 29)
Week 3 (*n* = 648)	117	18.1 (15, 21)
Week 4 (*n* = 642)	94	14.6 (12, 17)

^a^Samples were collected separately for each breast

^b^Milk collected from women who reported mastitis at the time of collection or the day before milk collection

Breast milk samples from women who did not report mastitis at weeks 1, 2, 3 and 4 postpartum were also cultivated for *S. aureus* (Table 3). Data are presented as *S. aureus* positive milk samples collected per week. Thirty two percent (207/657) of the milk samples collected at week 1 were culture positive for *S. aureus.* Culture positive milk samples at weeks 2, 3 and 4 were 26 % (172/660), 18 % (117/648) and 15 % (94/642) respectively. *S. aureus* was isolated more frequently in milk samples collected from women who reported mastitis at the time, or the day before milk collection, than from milk samples collected when women did not report mastitis (Table 3).

## Discussion

### Main findings

During the eight week study period, 20 % of participants developed mastitis; the majority of episodes were reported in the first four weeks postpartum. Women had an increased risk of developing mastitis if they reported nipple damage, problems with attachment, an over-supply of breast milk, nipple shield use or if they reported expressing breast milk several times a day. The presence of *S. aureus* on the nipple or in breast milk samples also increased the risk of developing mastitis using a prospective study design.

### Strengths and limitations

A strength of this study is that participants had not previously breastfed or experienced mastitis; they were recruited prior to starting breastfeeding. Some studies have suggested that women who have previously experienced mastitis are more likely to experience this condition again with subsequent infants [13, 14, 35]. Our cohort was followed until eight weeks postpartum with milk samples, breast and nipple analysis and detailed questionnaires collected concurrently at defined time-points, facilitating a comprehensive investigation of the development of mastitis in the early weeks postpartum. We also collected oral and nasal samples from infants to investigate whether infant colonisation with *S. aureus* could represent a potential route of transmission contributing to the development of mastitis. The prospective data collection enabled us to conduct a novel time-to-event analysis, whereas most studies of mastitis have used retrospective data collection [21, 24].

A limitation of this study is that we relied on maternal self-report for mastitis. We tried to overcome this by using a strict definition of mastitis; two breast symptoms and one systemic symptom, or treatment of mastitis with antibiotics. We also relied on self-report for problems with breastfeeding such as an over-supply of breast milk, problems with attachment and nipple shield use. Further, although we followed the cohort until eight weeks postpartum, we have microbiological data only to four weeks postpartum and so we were unable to carry out microbial assessments after this time.

### Interpretation

We used a strict definition of mastitis in this study to estimate the proportion of breastfeeding women who experienced a clinically significant illness. To reduce bias, we did not ask participants about mastitis directly. Using our definition, 20 % of the study population experienced at least one episode of mastitis, which is in agreement with several other Australian studies [1, 2, 36, 37]. However, in contrast to other studies [1, 17], mastitis was not more prevalent among private patients than women receiving public hospital care. This may be due to our small sample size or the fact that women in our study were followed only until eight weeks postpartum unlike previous studies where participants were followed-up at three months [17] and six months [1] after birth. It has been shown that most episodes of mastitis occur in the early weeks postpartum [1, 2]. In accordance with this, approximately three-quarters of the mastitis episodes occurred in the first four weeks postpartum in this study. Episodes of mastitis peaked at two weeks postpartum and decreased steadily after this point.

We investigated factors that could be correlated with the development of mastitis. We confirmed the important role of nipple damage as well as problems with attachment, over-supply of breast milk and use of a nipple shield in the development of mastitis. Since over 90 % of participants used nipple creams we did not include this in our analysis [31]. It is clear that damage to the nipple can contribute to mastitis [1–3, 12, 38]. Nipple damage occurs most frequently in the early postpartum period, suggesting that trauma to the nipple provides a portal of entry for microorganisms from the damaged skin into the nipple, leading to the development of mastitis [1, 3, 38]. Preventing nipple trauma and improving the management of damaged nipples could reduce the risk of developing mastitis among breastfeeding women. We confirmed previous reports that attachment problems are also associated with the development of mastitis [12]. It may be that incorrect attachment leads to nipple trauma which then contributes to the development of mastitis. Further, the fact that nipple shield use was associated with mastitis in our population suggests that some women who had nipple trauma used nipple shields to feed.

Participants who indicated that they had an over-supply of breast milk were also more likely to develop mastitis. We postulate that mastitis is more likely to occur in women with an over-supply of breast milk as they may experience more engorgement and milk stasis, which are known contributors to the development of mastitis [12, 14]. This study adds to the evidence that expressing several times each day is correlated with mastitis. Women who reported any expressing were not more likely to experience mastitis, however women who reported expressing several times a day had a higher risk of developing mastitis. Women may be using regular expressing to manage nipple trauma or an over-supply of milk. Possibly, the increased practice of regular expressing [20, 39], may be exacerbating over-supply and the risk of mastitis in some cases.

This study also investigated the role of *S. aureus* in the development of mastitis. Although nasal carriage of *S. aureus* plays a role in some infections [40], this study, as well as the previous study by Amir *et al*. found no association between maternal nasal carriage of this bacteria and mastitis [24]. Women who were culture-positive for *S. aureus* on their nipples or in their expressed breast milk also had an increased risk of developing mastitis. As *S. aureus* is the usual pathogen associated with mastitis in breastfeeding women [22–24] we also investigated the presence of *S. aureus* in breast milk from women with and without mastitis. Milk samples collected from women who reported mastitis at the time of, or the day prior to milk collection were more likely to grow *S. aureus* compared to samples from women without mastitis. This is in agreement with previous reports [22–24], and highlights the role of this species in the development of mastitis. Interestingly, many milk samples from healthy women were also positive for *S. aureus*, confirming the presence of this organism as a commensal on the skin and in the milk of healthy women who did not develop mastitis [41]. As Ingman et al. explained: “the interactions between inflammatory stimuli, including pathogenic bacteria, and other components of the microbiome, as well as the host immune response, are all likely to contribute to shaping the severity of mastitis, duration of symptoms and resolution of the disease” ([42] p. 1).

It is clear that mastitis impacts negatively on women’s lives; participants reported that their mastitis symptoms interfered with breastfeeding and with activities of daily living. In accordance with a previous study [5], participants reported that their symptoms had a greater impact on activities of daily living than on breastfeeding. The fact that mastitis has such a negative influence on women’s lives underlines the need to investigate factors that could be varied to reduce or prevent mastitis. The prevention or early treatment of damaged nipples, active management of breast engorgement and early intervention to help with positioning and attachment may potentially reduce the risk of mastitis in breastfeeding women.

## Conclusion

Twenty percent of CASTLE study participants developed mastitis in the first eight weeks postpartum and this had a negative impact on their lives. Nipple damage, problems with attachment, over-supply of breast milk, expressing several times a day, use of a nipple shield and *S. aureus* presence on the nipple or in expressed breast milk increased the risk of developing mastitis in our study sample. Education on optimal positioning and attachment leading to a reduction in nipple damage may reduce the incidence of mastitis among breastfeeding women [43].
